# Effect of Whey Protein Supplementation on Inflammatory and Antioxidant Markers, and Clinical Prognosis in Acute Ischemic Stroke (TNS Trial): A Randomized, Double Blind, Controlled, Clinical Trial

**DOI:** 10.15171/apb.2020.018

**Published:** 2019-12-11

**Authors:** Mazyar Hashemilar, Mohammad Khalili, Nasim Rezaeimanesh, Elyar Sadeghi Hokmabadi, Sevin Rasulzade, Seyed Morteza Shamshirgaran, Aliakbar Taheraghdam, Mehdi Farhoudi, Sheyda Shaafi, Seyed Kazem Shakouri, Daryoush Savadi Osgouei

**Affiliations:** ^1^Department of Neurology, School of Medicine, Tabriz University of Medical Sciences, Tabriz, Iran.; ^2^Neurosciences Research Center, Tabriz University of Medical Sciences, Tabriz, Iran.; ^3^Multiple Sclerosis Research Center, Neuroscience Institute, Tehran University of Medical Sciences, Tehran, Iran.; ^4^Healthy Aging Research Center, Neyshabur University of Medical Sciences, Neyshabur, Iran.; ^5^Physical Medicine and Rehabilitation Research Center, Tabriz University of Medical Sciences, Tabriz, Iran.

**Keywords:** Inflammation, Stroke, Brain ischemia, Malnutrition, Oxidative stress, Whey protein

## Abstract

***Purpose:*** Malnutrition is extensively prevalent amongst critically ill patients afflicted by ischemic stroke (IS). This study purpose was to evaluate the protein whey effect on inflammatory and antioxidant markers and functional prognosis in acute IS patients.

***Methods:*** out of 42 patients with acute IS who were referred to Imam Reza Educational Hospital, Tabriz, Iran, 40 patients participated in the study. Twenty-one patients as control group received the hospital routine formula, and 19 patients as intervention group received 20 g/daily of whey protein through oral gavage. Inflammation and oxidative stress indicators (e.g., albumin, malondialdehyde (MDA), total antioxidant capacity (TAC), interleukin-6 (IL-6), tumor necrosis factor alpha (TNF-α), and high sensitivity C reactive protein (hs-CRP)and clinical variables included in were evaluated using National Institutes of Health Stroke Scale (NIHSS) and modified Rankin Scale (mRS) during admission and also 3 weeks after intervention.

***Results:*** Whey protein supplementation significantly decreased the NIHSS and mRS scores, TNF-α, IL-6, and hs-CRP by passing 3 weeks from intervention (*P*<0.05). However, whey formula had no significant effect on other markers including albumin, and MDA. The hs-CRP (*P* = 0.02) reduction was significantly higher in whey protein group in comparison with control group.

***Conclusion:*** Whey protein supplementation reduced inflammation markers in those patients with IS. However, these changes should be studied in larger-scale trials.

## Introduction

By being the second most frequent mortality cause and also the first leading cause of adult disabilities, stroke is taking its toll in all over the world.^1^ In tandem with its global trend, the stroke prevalence was demonstrated to be very high in Iran by involving approximately 139 per 100 000 cases in 2006.^2^

The main mechanisms are disruption of cerebral blood flow and hypo-oxygenation, by which brain tissue is affected during an ischemic attack. This results in oxidative stress due to overproduction of reactive oxygen species,^3^ and also causes an imbalance between pro-oxidants and anti-oxidation defense system.^4^ Consequently, increased oxidative stress contributes to mitochondrial respiration dysfunction, pathologic increase in cytosolic calcium and also cytosolic pH acidification, which all of them result in death of neuronal cell.^5^ It has been indicated that oxidative stress biomarkers like malondialdehyde (MDA) and glutathione (GSH) were dramatically increased in patients with acute ischemic stroke (IS), however serum MDA level has been indicated to be associated to the size of infarct, score of Canadian neurological scale, and clinical outcomes.^6,7^ Inflammation is another factor that plays a crucial role in IS-induced ischemic cell death within the penumbra.^8^ Evidence proposes that inflammation peripheral biomarkers like interleukin-6 (IL-6), plasma C-reactive protein (CRP) concentration, and white blood cell count are associated with severity of acute IS, and clinical outcomes.^9^

Patients with IS frequently suffer from malnutrition, which is exacerbated by stroke-induced dysphagia.^10^ Evidence strongly supports that post-IS protein-energy malnutrition is a risk factor for deprived clinical outcomes.^3^ These patients usually express increased oxidative stress and inflammation levels, which contribute to their poor clinical outcomes, and if this condition was reversed, it could restore the protein anabolism/catabolism balance and benefit the critically ill ischemic patient.^11^

Whey protein possesses both antioxidant and anti-inflammatory characteristics as one of the richest sources of branched-chain amino acids like leucine. The Tabriz Nutritional Stroke trial was designed in order to investigate the supplementation with whey protein effect on the levels of inflammatory and antioxidant factors along with clinical prognosis in patients with IS.

## Materials and Methods

### Study design and patients

**Figure 1:**
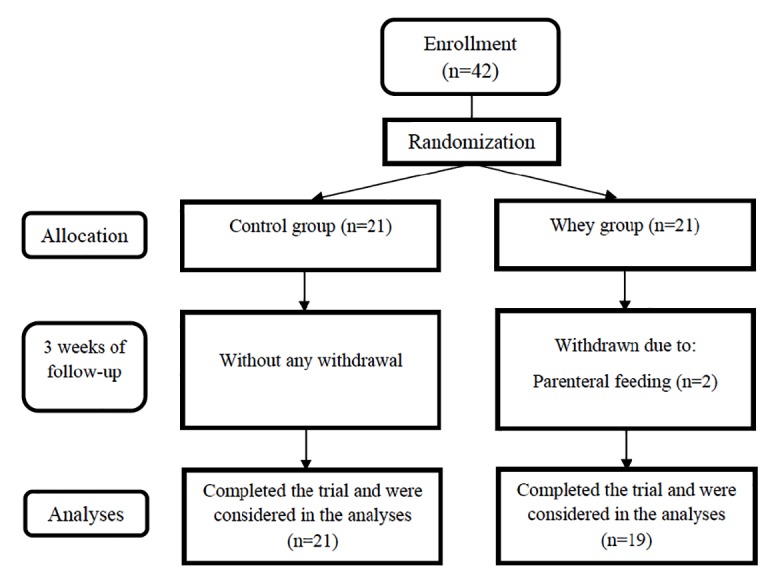
Flow diagram of the patients.

Forty-two patients with first-episode acute IS were randomly included in a randomized, double blind, controlled, clinical trial (Figure 1). The participants were selected from those patients who were referred to Imam Reza hospital of Tabriz University of Medical Sciences, Tabriz, Iran from February 2016 to August 2018. The patients were Tabriz stroke registry project participants.

### Inclusion and exclusion criteria

All the patients with the following criteria were included in this study in the respected period;

Those who had the age more than 40 years old, who were diagnosed with acute IS definitely, who were the first-ever IS patients, who had the National Institutes of Health Stroke Scale (NIHSS) scores less than 20, and those for whom the enteral feeding was accomplished in less than 48 hours after admission. On the other hand, patients who had one or more than one of the following features were excluded from this study: being in vegetative state, tissue plasminogen activator administration, chronic illnesses presence (chronic renal failure requiring dialysis, hepatic failure and cirrhosis, epilepsy, cancer, gastrointestinal bleeding, uncontrolled diabetes mellitus, history of heart attacks three months before the study, chronic respiratory diseases, heart failure, and hematologic illnesses), change in diagnosis, parenteral feeding, enteral feeding for less than ten days, treatment with immunosuppressive medications, alcohol and drug abuse, lactose intolerance, and death at the time of admission first 10 days.

### Study method

Demographic data of participants were collected. A neurologist initially evaluated all participants in order to investigate inclusion and exclusion criteria for them. The impairment as a result of stroke and disability rate were determined using NIHSS score, and the modified Rankin Scale (mRS), respectively.

NIHSS is a systematic method, which provides a quantitative measure of stroke-induced neurological damage and is generally was designed for those patients with acute stroke. The scale contains 15 items assessing the acute stroke effect on the consciousness, language, eye movements, motor force, ataxia, and sensorium level. Each item is scored either on a 3 or 5 point scale, zero indicates the normal state, and upper limit is 42.^12^ In addition, mRS scale contains 6 levels and evaluates independence instead of the specific tasks performance. The scale grades range from 0 to 6; zero signifies ‘with no symptoms’, five signifies ‘with severe disability’ and 6 indicates ‘death’. Sulter et al^13^ have extensively discussed on these grades.

Patients were assigned into intervention (n = 21) or control (n = 21) groups by random using Minitab software and also randomized block design (Figure 1). In the study duration, 2 patients were excluded from intervention group as a result of using parenteral nutrition, and finally statistical analyses were accomplished on 40 patients (19 intervention and 21 control participants). Patients’ randomization was performed by an experimenter who was blind to this study and grouping nature.

After that, patients in the control group received routine hospital feeding that was kitchen-made, containing 25 kcal/kg energy and 1.5 g/d protein per day. However, the intervention group participants received 20 gr of their daily protein from whey protein. In regard with that, clinical variables included in NIHSS and mRS and biochemical variables were evaluated at this study beginning and also by passing three weeks from intervention. A peripheral blood sample was attained from both groups’ participants (5 cc) and was centrifuged at 4000 rpm for 15 minutes. After that, the serum was isolated and stored for further analyses in labeled microtubes at a volume of 1 cc at -80°C in a freezer. Total albumin, oxidative stress markers (including total antioxidant capacity [TAC] and MDA), and inflammation indices (including IL-6, tumor necrosis factor alpha [TNF-α], and high sensitivity C-reactive protein [hs-CRP]) were measured by the use of appropriate kits and in terms of the manufactures’ instructions.

In this study, albumin, hs-CRP, TNF-α, IL-6, MDA, and TAC were evaluated as primary variables and gender, comorbidity history, smoking status, clinical severity (including NIHSS, and mRS) were evaluated as secondary variables.

### Statistical analyses

The IBM SPSS^®^ software version 16 was utilized in order to perform all statistical analyses. Data were expressed as mean ± standard deviation (SD), frequency, and percentage. Data Normal distribution was evaluated using Kolmogorov-Smirnov test. Also, chi-square test and Fisher’s exact test were applied for comparing the qualitative variables between the groups. In addition, independent *t* test and Mann-Whitney U test were utilized in order to compare quantitative variables. Moreover, paired *t* test or Wilcoxon signed-rank test was also used for examining intra-group changes before and after the intervention. *P*values≤0.05 were considered as statistically significant.

Sample size was calculated by the use of the formula that was stated in de Aguilar-Nascimento et al’s^11^ study. The power, acceptable error rate, and the drop probability were considered as 80%, 5%, and 20%, respectively.

## Results

Finally 19 IS patients in intervention group and 21 IS cases in control group were included in the present study, with respect to the inclusion and exclusion criteria. The participants mean ages were 65.73 years old and 71.90 years old in intervention group and control group, respectively. 89.5% of patients in intervention group and 81.0% of control group were male. The participants’ demographic and clinical data are indicated in Table 1. No significant differences were observed between these two study groups variables.

**Table 1:** Demographic and clinical data of the included participants

**Variables**	**Group**		***P*** **value**
	**Intervention (n=19)**	**Control (n=21)**	
Age (y)	65.73±11.63 (40-83)	71.90±5.91 (59-82)	0.15
Sex			0.66
Male	17 (89.5%)	17 (81.0%)	
Female	2 (10.5%)	4 (19.0%)	
History of comorbidity		0.23
Cardiovascular	4 (21.1%)	4 (19.1%)	
Hypertension	13 (68.4%)	13 (61.9%)	
Hyperlipidemia	2 (10.5%)	3 (14.2%)	
Diabetes	2 (10.5%)	3 (14.2%)	
Smoking			0.43
Yes	5 (26.3%)	8 (38.1%)	
No	14 (73.9%)	13(61.9%)	
Clinical severity			
NIHSS	7.36±4.28	5.28±4.57	0.07
MRS	1.94±0.97	1.85±1.49	0.32

Data are expressed as mean ± standard deviation (SD), frequency, and percentage.

Data analyses in control group demonstrated that the levels of IL-6, NIHSS, and mRS were significantly lower by passing 3 weeks from this study in comparison with the beginning of the study (*P*values were <0.01, <0.01, and 0.04, respectively). However, other variables indicated no significant changes in this group at the beginning and the end of the study (Table 2).

**Table 2:** Comparison of the parameters studied at the beginning and the end of the study in patients in the control group

**Variable**	**Evaluation time**		***P*** **value**
	**At the beginning**	**Three weeks later**	
Albumin (g/dL)	3.21±0.78	1.07±3.41	0.41
hs-CRP (µg/ml)	3.52±1.55	1.43±3.41	0.11
TNF-α (pg/mL)	18.11±13.14	9.02±15.94	0.20
IL-6 (pg/mL)	7.31 ± 5.81	4.41±3.87	<0.01
MDA (mmol/L)	1.81±0.31	0.38±1.82	0.88
TAC (µmol/L)	3835.14±881.8	938.21±3842.19	0.91
NIHSS	5.28±4.57	3.81±3.74	<0.01
MRS	1.85±1.49	1.47±1.21	0.04

Data are expressed as mean ± standard deviation (SD). hs-CRP, high sensitivity C-reactive protein; TNF, tumor necrosis factor; IL, interleukin; MDA, malondialdehyde; TAC, total antioxidant capacity.

As it was presented in Table 3, three weeks supplementation with whey protein in intervention group resulted in a significant reduction in hs-CRP (*P* value <0.01), TNF-α (*P* value = 0.02), NIHSS (*P* value <0.01), and mRS (*P* value = 0.02).

**Table 3:** Comparison of the parameters studied at the beginning and the end of the study in patients in the intervention group

**Variable**	**Evaluation time**		***P*** **value**
	**At the beginning**	**3 weeks later**	
Albumin (g/dL)	3.07±0.64	3.23±0.59	0.11
hs-CRP (µg/mL)	4.37±0.90	3.92±0.87	<0.01
TNF-α (pg/mL)	19.36±11.44	16.36±9.18	0.02
IL-6 (pg/mL)	8.83±6.88	4.38±4.77	<0.01
MDA (mmol/L)	2.03±0.24	2.04±0.22	0.68
TAC (µmol/L)	4084.21±1069.72	4224.31±1299.19	0.31
NIHSS	7.36±4.28	5.31±3.72	<0.01
MRS	1.94±0.97	1.68±0.82	0.02

Data are expressed as mean ± standard deviation (SD). hs-CRP, high sensitivity C-reactive protein; TNF, tumor necrosis factor; IL, interleukin; MDA, malondialdehyde; TAC, total antioxidant capacity.

Intergroup comparison demonstrated that the reduction in hs-CRP level was significantly higher in the intervention group in comparison with the control group (*P* = 0.02). Other laboratory parameters including TNF-α, IL-6, TAC, and MDA or variables that were associated to the patients’ clinical prognosis were also included in NIHSS and mRS, which indicated no significant changes between this study two groups during the investigation (Table 4).

**Table 4:** Comparison of the parameter changes between control and intervention groups during the study

**Variable**	**Group**		***P*** **value**
	**Control**	**Intervention**	
Albumin (g/dL)	0.11±0.57	0.16±0.45	0.71
hs-CRP (µg/ml)	-0.11±0.31	-0.45±0.53	0.02
TNF-α (pg/mL)	-2.14±7.41	-2.99±5.37	0.67
IL-6 (pg/mL)	-5.30±3.43	-6.56±4.44	0.61
MDA (mmol/L)	0.009±0.28	0.001±0.008	0.99
TAC (µmol/L)	7.90±51.42	140.11±585.91	0.33
NIHSS	-1.47±1.87	-2.05±1.92	0.34
MRS	-0.38±0.81	-0.26±0.45	0.56

Data are expressed as mean ± standard deviation (SD). hs-CRP, high sensitivity C-reactive protein; TNF, tumor necrosis factor; IL, interleukin; MDA, malondialdehyde; TAC, total antioxidant capacity.

## Discussion

We found that dietary three-week supplementation with whey protein caused higher reduction in hs-CRS serum levels in intervention group patients with IS, in comparison with the control group; however, we could not find any similar result in clinical findings, and also in association with other oxidative stress and inflammatory factors.

Recently, therapeutic strategies that reduce oxidation and inflammation have achieved considerable attention.^11,14^ It has been established that whey protein administration could reduce inflammation and oxidative stress in elderly patients with IS.^11,15^ However, this study assessed the whey protein affection on albumin, inflammatory and antioxidant markers, and also clinical prognosis amongst acute IS patients.

In a study accomplished by de Aguilar-Nascimento et al, the whey protein administration effects on IS patients were evaluated, and also were compared with those of casein-enriched diet. Their study results indicated a similar mortality rate in both groups that were associated with high serum levels of IL-6 and CRP. In addition, this study demonstrated that serum IL-6 was diminished and GSH levels escalated only in the whey protein-receiving group. Consequently, serum IL-6 was found to be lower, and GSH was indicated to be higher in whey protein-enriched IS patients in comparison with the casein group.^11^ These findings were in agreement with the those results achieved from this study indicating that whey protein supplementation could decrease inflammation in acute IS patients. In another study accomplished by Sheikholeslami Vatani and Ahmadi Kani Golzar, the anti-oxidative stress effects of whey protein administration with and without a 6-week exercise course were evaluated on over-weight individuals (body mass index: 25-30 kg/m^2^). This study demonstrated that whey protein administration along with exercise performing has a superior effect in comparison with the effect of exercise alone on the TAC reduction and increasing the levels of GSH.^16^ These findings were inconsistent with the results of this study, which could be due to different duration of two studies. Another study accomplished by Petyaev et al assessed the supplementation with whey protein lysosome formulation effects on inflammation and oxidative stress markers in those patients with prehypertension. This study indicated that this formula administration in patients with prehypertension exerted an almost two-fold decline in CRP levels and also reduced the inflammatory oxidative damage levels.^17^ However, Pal and Ellis could not show the anti-oxidative stress and anti-inflammatory effects of whey protein supplementation on obese individuals in comparison with control and casein groups.^18^ Similarly, Lee et al indicated that 12-week supplementation with whey protein had no significant effect on the inflammatory markers like IL-6 and CRP.^19^ A recent meta-analysis assessed the whey protein supplementation effects on serum CRP levels. A slight, but non-remarkable reduction was also found in CRP level with whey protein supplementation in the included participants. However, with respect to the nature of mentioned studies, a rather high heterogeneity was found amongst them, which could be caused by the different dose of whey used in them. Further analyses indicated that whey administration significantly reduced CRP levels in studies with a daily whey dose ≥20 g/d. In addition, it was found that the baseline CRP level in the patients was a potential modifier effect of whey protein administration in CRP levels diminishing.^20^

Generally, the differences observed in these studies could be caused by several factors including small sample size, variations in dose and duration of whey protein administration, and use of other therapeutic options along with whey, but it was not limited to them.

### Limitations and strengths

This study has several shortfalls including a limited number of patients, and assessing the whey supplementation effects for a short period.

On the other hand, it has several strengths as followings: As a clinical trial, this study enjoyed randomization, double blindness, and control group existence. Moreover, the changes in inflammatory markers during treatment may be caused by improvement of normal stroke inflammation that we made through analyzing time and treatment interaction in order to clarify the supplementation effects. In addition, our study investigated the stroke patients’ clinical prognosis under protein whey supplementation.

## Conclusion

In conclusion, this study results indicated the anti-inflammatory and clinical improvement effects of whey protein on acute IS patients, which could improve their prognosis in the long run. However, these results should be validated in larger-scale investigations with large sample sizes and long-term duration of interventions.

## Ethical Issues

Written informed consent was achieved from all of the included participants or from their family members. This study was accomplished following the approval of the Ethics Committee of Tabriz University of Medical Sciences (TBZMED.REC.1395.53) and was registered on the site for Iranian Registry of Clinical Trials under IRCT2016061428450N1 code.

## Conflict of Interest

The authors declare no conflict of interest.

## Acknowledgments

This study was financially supported by the Vice-Chancellor for Research of Tabriz University of Medical Sciences, Tabriz, Iran.
